# A novel model-based approach for dose determination of glycopyrronium bromide in COPD

**DOI:** 10.1186/1471-2466-12-74

**Published:** 2012-12-08

**Authors:** Helen Arievich, Tim Overend, Didier Renard, Michael Gibbs, Vijay Alagappan, Michael Looby, Donald Banerji

**Affiliations:** 1 Medars GmbH, Berlin, Germany; 2 Novartis Horsham Research Centre, Wimblehurst Road, Horsham, West Sussex, RH12 5AB, UK; 3 Novartis Pharma AG, Basel, Switzerland; 4 Novartis Pharmaceuticals Corporation, East Hanover, NJ, USA

**Keywords:** Glycopyrronium bromide (NVA237), Once-daily, Twice-daily, COPD, Dose–response, LAMA, Adherence

## Abstract

**Background:**

Glycopyrronium bromide (NVA237) is an inhaled long-acting muscarinic antagonist in development for treatment of COPD. This study compared the efficacy and safety of once-daily (OD) and twice-daily (BID) glycopyrronium bromide regimens, using a novel model-based approach, in patients with moderate-to-severe COPD.

**Methods:**

Double-blind, randomized, dose-finding trial with an eight-treatment, two-period, balanced incomplete block design. Patients (smoking history ≥10 pack-years, post-bronchodilator FEV_1_ ≥30% and <80% predicted, FEV_1_/FVC <0.7) were randomized to one of 16 independent sequences for 28 days. Primary endpoint: mean trough FEV_1_ at Day 28.

**Results:**

385 patients (mean age 61.2 years; mean post-bronchodilator FEV_1_ 53% predicted) were randomized; 88.6% completed. All OD and BID dosing regimens produced dose-dependent bronchodilation; at Day 28, increases in mean trough FEV_1_ versus placebo were statistically significant for all regimens, ranging from 51 mL (glycopyrronium bromide 12.5 μg OD) to 160 mL (glycopyrronium bromide 50 μg BID). Pharmacodynamic steady-state was reached by Day 7. There was a small separation (≤37 mL) between BID and OD dose–response curves for mean trough FEV_1_ at steady-state in favour of BID dosing. Over 24 hours, separation between OD and BID regimens was even smaller (FEV_1_ AUC_0-24h_ maximum difference for equivalent daily dose regimens: 8 mL). Dose–response results for FEV_1_ at 12 hours, FEV_1_ AUC_0-12h_ and FEV_1_ AUC_0-4h_ at steady-state showed OD regimens provided greater improvement over placebo than BID regimens for total daily doses of 25 μg, 50 μg and 100 μg, while the reverse was true for OD versus BID regimens from 12–24 hours. The 12.5 μg BID dose produced a marginally higher improvement in trough FEV_1_ versus placebo than 50 μg OD, however, the response at 12 hours over placebo was suboptimal (74 mL). Glycopyrronium bromide was safe and well tolerated at all doses.

**Conclusions:**

Glycopyrronium bromide 50 μg OD provides significant bronchodilation over a 24 hour period, and in terms of FEV_1_ AUC_0-24h_ is not significantly different than the same total daily dose administered BID. Importantly, OD dosing may confer better patient adherence. The results are consistent with previous glycopyrronium bromide studies and support once-daily dosing of glycopyrronium bromide 50 μg in patients with moderate-to-severe COPD.

**Trial registration:**

ClinicalTrials.gov: NCT01119950

## Background

Chronic obstructive pulmonary disease (COPD) is characterized by progressive airflow obstruction, resulting in airflow limitation that is only partially reversible [1,2]. COPD is a major cause of morbidity and mortality worldwide, and represents a substantial economic and social burden that is expected to increase [1].

Bronchodilators are the cornerstone treatment for all COPD severity stages. Long-acting muscarinic antagonists (LAMAs) are one of the main classes of bronchodilators used for the treatment of COPD. The optimal characteristics of a LAMA for use in COPD would be to provide the following: clinically meaningful bronchodilation, in addition to symptom relief, prevention of disease progression, improvement in exercise tolerance and health status, prevention and treatment of complications and exacerbations and a reduction in mortality risk [1,3,4]. Other characteristics of an ideal LAMA would include strong selectivity for muscarinic type 3 (M3) receptors, long duration of action, rapid onset of action, and a good safety and tolerability profile [1,4].

NVA237 is a dry-powder formulation of glycopyrronium bromide. As with other LAMAs, its bronchodilatory effects result from blockade of muscarinic type 1 (M1) and M3 receptors, which are involved in either transmission of nerve impulses or promotion of contraction in airway smooth muscle. Glycopyrronium bromide has a low oral bioavailability, is selective for M3 over muscarinic type 2 (M2) receptors, and equilibrates rapidly with M3 receptors [4]. Dosages of 50 μg and 100 μg once daily (OD) have been identified in a phase II trial as providing effective bronchodilation, with clinically meaningful improvements in forced expiratory volume in 1 second (FEV_1_) over 7 days’ treatment. Compared with open-label tiotropium 18 μg OD, glycopyrronium bromide 50 μg OD appears similar in terms of bronchodilatory efficacy and duration of action, although the latter has a more rapid onset of action [5,6]. Post-hoc analysis has also confirmed superior bronchodilation and responder rates with glycopyrronium bromide 50 μg, compared with lower doses (12.5 and 25 μg), while tolerability profiles were similar across the dose range [7]. The efficacy of glycopyrronium bromide 50 μg OD has been confirmed in a further study, showing it to provide sustained 24-hour bronchodilation during 14 days’ treatment [8]. In these studies, glycopyrronium bromide was well tolerated, even at dosages as high as 200 μg OD (4 times the dosage identified as the lowest dose producing clinically significant improvements in bronchodilation) [9]. Based on the results of these earlier efficacy and safety studies, a dose of 50 μg OD was selected for further clinical evaluation in Phase III trials.

The present study in patients with stable COPD aimed to evaluate the efficacy and safety of twice-daily (BID) dosing regimens for glycopyrronium bromide, versus the same total daily dose administered on an OD basis, by fully characterizing the dose–response relationship of both regimens. The doses evaluated ranged from 12.5 μg OD to 50 μg BID. Standard statistical methods routinely applied in bronchodilator studies, such as analysis of covariance, are intended to allow direct pairwise comparisons. However, these methods are not suitable for detailed dose–response characterization because they only provide information on each comparison independently and not on the dose–response relationship as a whole. Given the number of pairwise comparisons necessary to piecewise reconstruct a dose–response relationship with reasonable resolution, none of the pairwise comparisons will have adequate precision to allow differentiation across the doses due to sample size limitations. This fundamental limitation in dose–response assessment for spirometric measures has been discussed previously [10,11]. For these reasons, a rigorous pre-specified model-based approach was used to allow definitive estimation of the dose–response and dose–regimen relationships. This relationship, in turn, was used as the basis for dose differentiation.

## Methods

This study was conducted between April and December 2010 at 50 centres in 9 countries (Belgium, Germany, Hungary, India, The Netherlands, Poland, Romania, Spain and USA). The study was performed according to the ethical principles of the Declaration of Helsinki; the protocol was reviewed by the Independent Ethics Committee or Institutional Review Board for each participating centre. All patients provided written informed consent before enrolling in the study (Additional file 1: Appendix 1).

### Patients

Included in this study were male and female adults aged ≥40 years with stable moderate-to-severe COPD [12] and a smoking history of ≥10 pack-years (defined as: 20 cigarettes/day for 10 years, or 10 cigarettes/day for 20 years, etc.). Patients were required to have a post-bronchodilator (45 min after inhalation of 84 μg ipratropium bromide) FEV_1_ ≥30% and <80% of the predicted normal, and post-bronchodilator FEV_1_ to forced vital capacity (FVC) ratio <0.7 at screening. Patients must also have been symptomatic, according to daily electronic diary data collected during a 1-week run-in period, with a total score ≥1 on at least 4 of the 7 days prior to Day 1 of the study.

Study exclusion criteria included: hospitalization for exacerbation of airways disease in the 6 weeks prior to study start, respiratory tract infection within 4 weeks prior to study start, prolonged QTc interval at screening (or history of long QT syndrome), requiring daily oxygen therapy for chronic hypoxemia, history of asthma, Type 1 or uncontrolled Type 2 diabetes, history of malignancy in the previous 5 years (except localized basal cell carcinoma of the skin), or any other clinically relevant medical condition or laboratory abnormality that might have compromised safety or compliance. Also excluded were those with history of an untoward reaction to any of the study drugs, women of child-bearing potential not using an accepted form of contraception, pregnant women, and nursing mothers.

Use of inhaled salbutamol/albuterol as rescue medication was permitted throughout the study (except immediately before and during study visits, unless absolutely necessary). Patients taking fixed combinations of inhaled corticosteroids (ICS) and long-acting β_2_-agonists (LABA) were transferred to the nearest equivalent ICS monotherapy at least 48 hours prior to screening. Use of the following medications was not permitted during this study: long-acting anticholinergics (minimum washout period prior to screening: 7 days), short-acting anticholinergics (8 h), LABAs (48 h), short-acting β_2_-agonists other than rescue medication (6 h), fixed combinations of inhaled short-acting anticholinergics and short-acting β_2_-agonists (8 h), xanthines (7 days), parenteral or oral corticosteroids (1 month), and intramuscular depot corticosteroids (3 months). Also prohibited were: any drugs with potential to significantly prolong the QT interval (e.g. mizolastin; minimum washout prior to screening: 14 days or 5 half-lives, whichever was longer), other investigational drugs (30 days or 5 half-lives, whichever was longer), non-selective beta-blockers (7 days), leukotriene antagonists (7 days), cromoglycate (7 days), Nedocromil (7 days), Ketotifen (7 days), systemic anticholinergics (7 days). Patients who had live attenuated vaccinations within 30 days prior to the screening visit or during the run-in period were excluded from taking part in the study (inactivated influenza vaccination, pneumococcal vaccination or any other inactivated vaccine was acceptable provided it was administered ≥48 hours prior to the screening and/or randomization visit).

### Study design

This was a double-blind, randomized, dose-finding trial utilizing an eight-treatment, two-period, balanced incomplete block design. Patients were randomized to one of 16 independent sequences and received two treatments from: glycopyrronium bromide 12.5 μg OD, 12.5 μg BID, 25 μg OD, 25 μg BID, 50 μg OD, 50 μg BID, 100 μg OD, or placebo in a 1:1:1:1:1:1:1:1 ratio, with balanced representation of each treatment. Patients received study drug for 28 days in the first treatment period, then entered a 7-day washout period, during which they continued only with their background COPD medication, before commencing the second 28-day treatment period. All short-acting COPD medications were thoroughly washed out prior to commencing the second treatment period. Patients received their first dose of study medication in the clinic between 08:00 and 10:00 in the morning. Patients were instructed to take study medication every day at home between 08:00 and 10:00 in the morning, and the evening dose 12 hours later (±15 min), between 20:00 (8 pm) and 22:00 (10 pm). For patients on OD regimens, active treatment was administered in the morning and placebo in the evening, to maintain blinding of OD versus BID regimens. Patients were required to attend the study centres for 18 visits.

### Study assessments and variables

Efficacy assessments were based on centralized spirometry (using standardized spirometry equipment with review of data by qualified personnel at a Contract Research Organization) FEV_1_ and FVC were measured on Days 1, 7 and 14 at the following timepoints relative to the morning dose: 45 and 15 minutes pre-dose and 5 minutes and 1, 2, 4, 8, 10 hours, 11 hours 55 minutes, and 14 hours post dose, with assessments continuing the following day at 20 and 22 hours, 23 hours 15 minutes and 23 hours 45 minutes post dose. Day 28 spirometry tests followed the same schedule, with additional assessments at 15 minutes and 3 and 6 hours post dose.

The primary efficacy variable was trough FEV_1_ at Day 28 (defined as the mean of the 23 h 15 min and 23 h 45 min post-dose values). Secondary efficacy variables included FEV_1_ area under the curve for time 0–24 hours post dose (AUC_0-24h_ FEV_1_), FEV_1_ AUC_0-4h_, FEV_1_ AUC_0-12h_, FEV_1_ AUC_12-24h_, FEV_1_ at 12 hours and peak FEV_1_, all measured after 28 days of treatment. The AUC measures were standardized by the length of time interval.

Safety assessments consisted of collecting adverse events (AEs; including their severity and possible relationship to study drug) and pregnancies, assessing vital signs, and electrocardiogram (ECG) throughout the study. Haematology, blood chemistry and urinalysis parameters were assessed at screening and at the final dosing visit in each treatment period. An independent adjudication committee was used to classify deaths during the study.

### Statistical analyses

A non-linear dose–response modelling approach was used to evaluate the bronchodilator efficacy of doses administered either OD or BID after 28 days of treatment (Additional file 2: Appendix 2). Based on previous glycopyrronium bromide spirometric results [5], the shape of the dose–response curve was assumed to be of E_max_-type:

(1) $$\frac{E_{\max}\times {\mathrm{dose}}^{\gamma }}{{\left(ED_{50}/\theta^{I\left(\mathrm{BID}\right)}\right)}^{\gamma }+{\mathrm{dose}}^{\gamma }}$$

where dose represents total daily dose, E_0_ is the placebo effect, E_max_ = the maximum (placebo-adjusted) drug effect, ED_50_ = a measure of drug potency interpreted as the dose providing 50% of the maximum effect and γ is a steepness parameter. Further assumptions were that regimen acts as a potency (ED_50_) modifier and that a common maximal effect (E_max_) is reached for both regimens. A sensitivity analysis of trough FEV_1_, performed relaxing the latter assumption, provided similar results.

For each spirometry outcome, the actual analysis used data collected over the entire course of treatment (Days 1, 7, 14 and 28). To reduce potential bias, eight candidate models were selected to characterize the bronchodilatory dose response over time and were prospectively specified. Half of the models had a fixed value γ=1 in the above equation; the other half allowed estimating the γ parameter. For each set of four models, the longitudinal component either assumed a smooth increase in FEV_1_ response from Day 1 to Day 28, or a flat response at Day 7 onwards. Each model included random terms to represent inter-individual (patient), inter-occasion (patient–period) and residual variability, all assumed to be normally distributed. Each model was further adjusted using the SAS procedure NLMIXED with the first-order method (method = FIRO) for: period baseline FEV_1_ measurement; FEV_1_ prior to inhalation and FEV_1_ 45 minutes post inhalation of ipratropium bromide (components of reversibility at Day 14); smoking status (current/ex-smoker); baseline ICS use (yes/no); and period (fixed effect).

Each of the candidate models that were successfully fitted provided an estimate of dose response at Day 28. The final characterization of dose response relied on model averaging, i.e. a weighted average of the different models. Weights for each model were a function of the Bayesian information criterion (BIC), i.e. models that best represented the data carried a greater weight in the estimate. Confidence limits around model-average estimates were obtained using a simulation-based procedure.

Results were expressed as percentages of the maximal placebo-adjusted effect of glycopyrronium bromide (E_max_) and treatment differences were summarized for OD and BID regimens that provided the same total daily dose. No formal hypothesis testing was performed for this study; however, 90% confidence intervals (CIs) are presented for selected analyses.

In order to characterize bronchodilation over the course of the whole day at steady-state, dose–response modelling was applied to the measured FEV_1_ for each spirometry timepoint separately (using the model assuming a flat dose response at Day 7 onwards and γ=1) and the results plotted to show the 24-hour profile.

The modified intent-to-treat (ITT) population included all those patients randomized who received at least one dose of study medication. These were used in the analysis of all efficacy variables. The safety population, used in the analysis of all safety variables, included all those patients who received at least one dose of study medication, including those who may have received it in error (this was identical to modified ITT population except for allowing for erroneous drug administration). Safety variables were analyzed descriptively, with clinically relevant or notable changes assessed. QTc interval was calculated using Fridericia’s formula: QTc = QT / ^3^√RR.

Assuming an overall dropout rate of 17%, a planned sample size of 360 patients was considered sufficient to show that the upper limit of the 90% CI (one-sided) for the maximum BID–OD difference in trough response falls below 40 mL with 80% chance, when the relative potency of BID versus OD regimens was assumed to be 120%.

## Results

### Patients

A total of 385 patients were randomized and received study medication, with 341 (88.6% of the modified ITT population) completing the study, providing evaluable data for 87–96 patients per treatment group. Baseline demographic and clinical characteristics are shown in Table 1. The mean age of patients in the study was 61.2 years (range 40–81 years, with the majority of patients being <65 years of age), 65.0% were male; and the mean duration of COPD was 7.7 years. Mean post-bronchodilator FEV_1_ was 53% predicted.

**Table 1:** Baseline demographics and clinical characteristics (safety population)

	**Glycopyrronium bromide 12.5 μg OD (n = 89)**	**Glycopyrronium bromide 25 μg OD (n = 96)**	**Glycopyrronium bromide 12.5 μg BID (n = 96)**	**Glycopyrronium bromide 50 μg OD (n = 92)**	**Glycopyrronium bromide 25 μg BID (n = 96)**	**Glycopyrronium bromide 100 μg OD (n = 96)**	**Glycopyrronium bromide 50 μg BID (n = 87)**	**Placebo (n = 91)**
Age (years), mean (SD)	60.2 (7.77)	60.0 (7.98)	60.9 (7.89)	59.2 (8.14)	61.2 (7.80)	62.1 (7.83)	62.2 (7.74)	63.2 (7.67)
Range	47-79	43-81	40-80	43-80	40-80	40-79	49-81	48-78
Sex, n (%)								
Male	56 (62.9)	64 (66.7)	67 (69.8)	56 (60.9)	66 (68.8)	59 (61.5)	59 (67.8)	56 (61.5)
Smoking history, n (%)								
Ex-smoker	45 (50.6)	51 (52.1)	50 (52.1)	45 (48.9)	50 (52.1)	57 (59.4)	48 (55.2)	52 (57.1)
Current smoker	44 (49.4)	45 (46.9)	46 (47.9)	47 (51.1)	46 (47.9)	39 (40.6)	39 (44.8)	39 (42.9)
Pack-years, mean (SD)	43.2 (23.05)	39.6 (19.97)	40.9 (19.88)	39.3 (19.91)	42.8 (23.26)	39.8 (19.79)	42.1 (21.17)	40.4 (20.84)
FEV_1_ pre-bronchodilator (% predicted), n (%)								
	47 (12)	46 (11)	46 (14)	46 (11)	45 (12)	46 (14)	48 (13)	48 (13)
FEV_1_ post-bronchodilator (% predicted), n (%)								
30- < 50%	34 (38.2)	36 (37.5)	44 (45.8)	34 (37.0)	43 (44.8)	44 (45.8)	34 (39.1)	37 (40.7)
50- < 80%	55 (61.8)	60 (62.5)	52 (54.2)	58 (63.0)	53 (55.2)	52 (54.2)	53 (60.9)	54 (59.3)
FEV_1_ reversibility,%								
Mean (SD)	17 (12)	19 (16)	16 (14)	17 (12)	16 (12)	17 (18)	16 (16)	16 (16)
Median (range)	16 (−11-44)	19 (−16-90)	16 (−43-55)	18 (−18-40)	15 (−13-44)	16 (−43-72)	14 (−21-90)	13(−21-72)
FEV_1_/FVC (%) post-bronchodilator (%), mean (SD)								
	46.87 (10.36)	46.76 (8.66)	45.86 (11.12)	46.41 (9.29)	46.15 (10.21)	47.21 (11.72)	47.89 (9.57)	48.22 (10.65)

OD: once daily; BID: twice daily; SD: standard deviation; FEV_1_: forced expiratory volume in one second; FVC: forced vital capacity.

### Efficacy

#### Primary endpoint: mean trough FEV_1_ at Day 28

**Figure 1:**
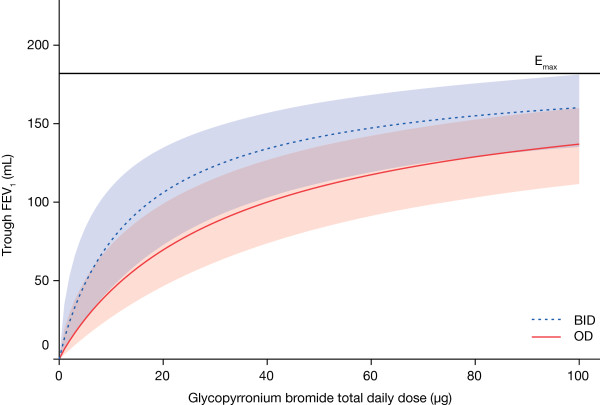
**Trough FEV**_**1**_**dose−response results at 24 hours at steady-state (modified intent-to-treat population).** Model average placebo-adjusted values for OD and BID dosing regimens, with 90% confidence limits (red and blue shaded areas, respectively). FEV_1_: forced expiratory volume in one second; OD: once daily; BID: twice daily.

Glycopyrronium bromide reached pharmacodynamic steady-state by Day 7. At Day 28, all once daily and twice daily dosing regimens produced dose-dependent bronchodilation (Figure 1). Furthermore, all glycopyrronium bromide treatment groups had statistically significant absolute increases in mean trough FEV_1_ compared with placebo. Increases in FEV_1_ over that seen with placebo ranged from 51 mL (with glycopyrronium bromide 12.5 μg OD) to 160 mL (with glycopyrronium bromide 50 μg BID), equating to 27-85% of the model-predicted maximum effect of any glycopyrronium bromide dose (Table 2). For the primary efficacy variable of mean trough FEV_1_, separation between dose–response curves for OD and BID dosing regimens was observed although the difference was small, with an estimated difference of approximately 35 mL, favouring BID, for total daily doses in the range of 25–50 μg (Figure 1).

**Table 2:** **Dose–response relationship for glycopyrronium bromide treatment regimens: model-averaged analysis of trough FEV**_**1**_**, FEV**_**1**_**AUC**_**0-24h**_**, FEV**_**1**_**AUC**_**0-12h**_**, FEV**_**1**_**AUC**_**0-4h**_**, and FEV**_**1**_**AUC**_**12-24h**_**at steady state**

	**Glycopyrronium bromide dose**						
	**12.5 μg OD**	**25 μg OD**	**12.5 μg BID**	**50 μg OD**	**25 μg BID**	**100 μg OD**	**50 μg BID**
*Trough FEV*_*1*_*at steady state*							
Absolute increase over placebo (L), (SE)	0.051 (0.019)	0.079 (0.020)	0.115 (0.021)	0.109 (0.020)	0.141 (0.020)	0.137 (0.019)	0.160 (0.020)
90% CI	0.032, 0.081	0.054, 0.108	0.082, 0.142	0.083, 0.135	0.112, 0.163	0.111, 0.160	0.135, 0.181
% of projected maximum effect of any glycopyrronium bromide dose* (SE)	27 (11)	42 (12)	62 (14)	59 (12)	76 (13)	73 (12)	85 (11)
90% CI	16, 44	27, 61	37, 82	38, 76	49, 90	49, 87	60, 95
*FEV*_*1*_*AUC*_*0-24h*_*at steady state*							
Absolute increase over placebo (L), (SE)	0.058 (0.012)	0.089 (0.014)	0.098 (0.015)	0.123 (0.014)	0.131 (0.014)	0.152 (0.012)	0.158 (0.012)
90% CI	0.039, 0.079	0.066, 0.113	0.071, 0.122	0.099, 0.145	0.106, 0.152	0.131, 0.171	0.138, 0.176
% of projected maximum effect of any glycopyrronium bromide dose* (SE)	29 (7)	45 (8)	49 (9)	62 (8)	66 (8)	76 (6)	79 (6)
90% CI	19, 42	32, 59	34, 64	48, 74	51, 78	65, 85	68, 88
*FEV*_*1*_*AUC*_*0-12h*_*at steady state*							
Absolute increase over placebo (L), (SE)	0.085 (0.015)	0.121 (0.016)	0.104 (0.016)	0.152 (0.014)	0.139 (0.015)	0.176 (0.012)	0.166 (0.013)
90% CI	0.061, 0.111	0.094, 0.146	0.078, 0.130	0.128, 0.174	0.113, 0.162	0.155, 0.195	0.144, 0.186
% of projected maximum effect of any glycopyrronium bromide dose* (SE)	41 (8)	58 (8)	51 (8)	74 (6)	67 (7)	85 (4)	80 (5)
90% CI	29, 55	45, 71	37, 64	62, 83	54, 78	76, 91	70, 88
*FEV*_*1*_*AUC*_*0-4h*_*at steady state*							
Absolute increase over placebo (L), (SE)	0.105 (0.016)	0.138 (0.015)	0.122 (0.016)	0.165 (0.013)	0.153 (0.014)	0.183 (0.012)	0.175 (0.012)
90% CI	0.080, 0.132	0.114, 0.162	0.096, 0.148	0.143, 0.185	0.129, 0.174	0.163, 0.203	0.154, 0.195
% of projected maximum effect of any glycopyrronium bromide dose* (SE)	51 (8)	67 (7)	59 (8)	80 (6)	74 (7)	88 (5)	84 (6)
90% CI	39, 64	54, 78	46, 72	67, 87	61, 83	78, 93	73, 91
*FEV*_*1*_*AUC*_*12-24h*_*at steady state*							
Absolute increase over placebo (L), (SE)	0.051 (0.025)	0.079 (0.026)	0.112 (0.027)	0.111 (0.026)	0.141 (0.026)	0.141 (0.027)	0.163 (0.028)
90% CI	0.033, 0.080	0.056, 0.108	0.080, 0.139	0.086, 0.137	0.112, 0.163	0.116, 0.163	0.138, 0.184
% of projected maximum effect of any glycopyrronium bromide dose* (SE)	26 (11)	40 (12)	57 (14)	56 (13)	71 (14)	71 (13)	82 (13)
90% CI	16, 43	24, 60	31, 78	33, 75	42, 88	43, 86	51, 94

*100 x (effect of this dosage/maximum theoretical effect predicted by model).

OD: once daily; BID: twice daily; SE: standard error; CI: confidence interval; FEV_1_: forced expiratory volume in one second; AUC: area under the curve.

#### Secondary endpoint: FEV_1_ AUC at Day 28

**Figure 2:**
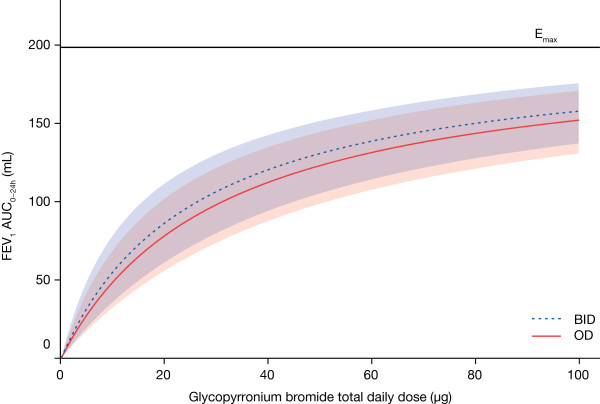
**Dose−response results of FEV**_**1**_**AUC**_**0-24h**_**at steady-state (modified intent-to-treat population).** Model average placebo-adjusted values for OD and BID dosing regimens, with 90% confidence limits (red and blue shaded areas, respectively). FEV_1_: forced expiratory volume in one second; AUC: area under the curve; OD: once daily; BID: twice daily.

When FEV_1_ over the whole 24-hour period post-morning dose was considered, the separation was substantially reduced, with OD and BID regimens showing similar dose–response profiles for FEV_1_ AUC_0-24h_ for equivalent total daily doses (Figure 2). The estimated maximum difference between OD and dose-equivalent BID regimens was 8 mL over 24 hours, at steady-state. Differences between the OD and BID regimens for the total daily doses of 25 μg, 50 μg and 100 μg were small (Table 2). For the 50 μg OD dose, the treatment difference over placebo was 123 mL (62% E_max_) compared with 131 mL (66% E_max_) for 25 μg BID and 98 mL (49% E_max_) for 12.5 μg BID.

Dose–response results for FEV_1_ AUC_0-4h_, FEV_1_ AUC_0-12h_, and FEV_1_ AUC_12-24h_ at steady-state were also comparable for OD and dose-equivalent BID regimens (Table 2). For the 50 μg OD dose the treatment difference for FEV_1_ AUC_0-4h_ was 165 mL (79.7% E_max_) versus 153 mL (73.7% E_max_) for 25 μg BID, and 122 mL (58.9% E_max_) for 12.5 μg BID. The treatment difference for FEV_1_ AUC_0-12h_ was 152 mL (73.6% E_max_) for 50 μg OD versus 139 mL (67.1% E_max_) for 25 μg BID, and 104 mL (50.5% E_max_) for 12.5 μg BID. Finally, for FEV_1_ AUC_12-24h,_ the treatment difference was 111 mL (56.4% E_max_) for 50 μg OD compared with 141 mL (71.4% E_max_) for 25 μg BID, and 112 mL (56.6% E_max_) for 12.5 μg BID (Table 2).

#### Secondary endpoint: peak FEV_1_ at Day 28

**Figure 3:**
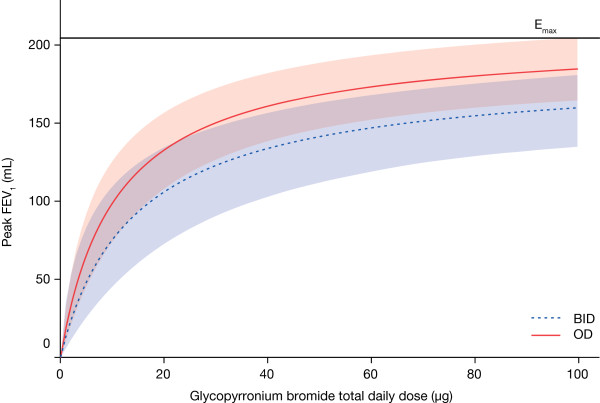
**Dose−response results of peak FEV**_**1**_**at steady-state (modified intent-to-treat population).** Model average values for OD and BID dosing regimens, with 90% confidence limits (red and blue shaded areas, respectively). FEV_1_: forced expiratory volume in one second; OD: once daily; BID: twice daily.

When peak FEV_1_ at steady-state was considered, as anticipated, an advantage was seen for the OD over the BID regimen (Figure 3). For the 50 μg OD dose the treatment difference was 168 mL (82% E_max_), compared with 156 mL (76% E_max_) for 25 μg BID and 126 mL (61% E_max_) for 12.5 μg BID.

#### Comparison of spirometric profiles

**Figure 4:**
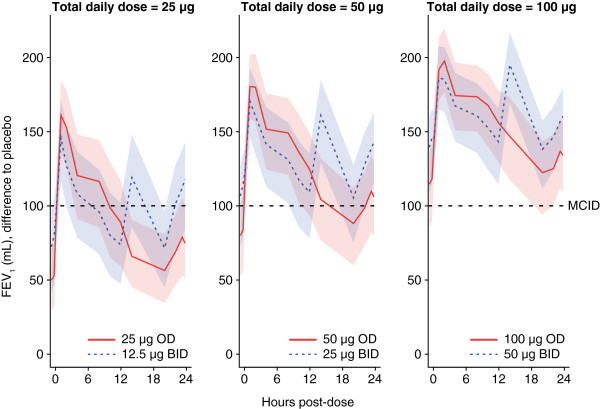
**24-hour spirometry results at steady-state (modified intent-to-treat population).** Model-based placebo-adjusted values for OD and BID dosing regimens, with 90% confidence limits (red and blue shaded areas, respectively). MCID = 100 mL [12]. MCID: minimal clinically important difference; FEV1: forced expiratory volume in one second; OD: once daily; BID: twice daily.

In general, improvements in spirometric outcomes based on measurements collected 0–12 hours post-dose (FEV_1_ at 12 h, FEV_1_ AUC_0-4h_ and FEV_1_ AUC_0-12h_) were marginally greater for the OD regimens than the BID regimens, whereas the opposite was seen over the period 12–24 hours post-dose. The reason for this can best be visualized through the full spirometry profiles, which are shown for total daily doses of 25, 50 and 100 μg in Figure 4. The dose response is apparent in these plots, as the profiles are shifted upwards with increasing total daily dose. As can also be seen, the OD profiles were above the BID profiles over the first 12 hours, and below over the last 12 hours. Thus, OD dosing shows more favourable results during the day and BID dosing more favourable results during the night. Over the whole day, those differences tend to cancel out which leaves no meaningful differences between OD and BID dosing in AUC_0-24h_ (Figure 2).

Figure 4 also shows that the glycopyrronium bromide 50 μg OD regimen is the lowest OD dose that provides bronchodilation above or around the minimal clinically important difference (MCID) [12]. Although the 12.5 μg BID dose produced a marginally higher improvement in trough FEV_1_ over placebo than the 50 μg OD dose (Day 28), the response it produced in FEV_1_ at 12 hours after 28 days of treatment over placebo (i.e. 74 mL) was below the MCID of 100 mL (the minimum difference that can be perceived by patients, and which correlates with fewer relapses following exacerbations) [13].

### Safety

The overall incidence of AEs was lower with all glycopyrronium bromide dosages/regimens than with placebo (Table 3). The most commonly reported AEs were: COPD (worsening) in the glycopyrronium bromide 12.5 μg OD and 25 μg BID groups; nasopharyngitis in the glycopyrronium bromide 25 μg OD, 50 μg OD and 50 μg BID groups; both nasopharyngitis and headache in the glycopyrronium bromide 100 μg OD and placebo groups; and dyspnoea and diarrhoea in the glycopyrronium bromide 12.5 μg BID group. However, differences in AE frequency between treatments were generally small and there was no difference in severity between the OD and BID regimens.

**Table 3:** Adverse events occurring during 28 days’ treatment with glycopyrronium bromide OD and BID treatment regimens

**n (%) patients**	**Glycopyrronium bromide 12.5 μg OD (n = 89)**	**Glycopyrronium bromide 25 μg OD (n = 96)**	**Glycopyrronium bromide 12.5 μg BID (n = 96)**	**Glycopyrronium bromide 50 μg OD (n = 92)**	**Glycopyrronium bromide 25 μg BID (n = 96)**	**Glycopyrronium bromide 100 μg OD (n = 96)**	**Glycopyrronium bromide 50 μg BID (n = 87)**	**Placebo (n = 91)**
Any AE	24 (27.0)	20 (20.8)	17 (17.7)	26 (28.3)	23 (24.0)	27 (28.1)	20 (23.0)	29 (31.9)
Any serious AE	2 (2.2)	2 (2.1)	1 (1.0)	3 (3.3)	4 (4.2)	3 (3.1)	1 (1.1)	3 (3.3)
Serious AE leading to discontinuation	2 (2.2)	1 (1.0)	1 (1.0)	1 (1.1)	2 (2.1)	2 (2.1)	0	2 (2.2)
Most common AEs by preferred term*								
Nasopharyngitis	4 (4.5)	8 (8.3)	2 (2.1)	4 (4.3)	3 (3.1)	4 (4.2)	5 (5.7)	6 (6.6)
COPD worsening	7 (7.9)	3 (3.1)	2 (2.1)	0	5 (5.2)	3 (3.1)	1 (1.1)	4 (4.4)
Headache	1 (1.1)	1 (1.0)	2 (2.1)	3 (3.3)	3 (3.1)	4 (4.2)	3 (3.4)	6 (6.6)
Dyspnoea	0	2 (2.1)	3 (3.1)	3 (3.3)	2 (2.1)	1 (1.0)	4 (4.6)	0
Cough	3 (3.4)	3 (3.1)	1 (1.0)	0	1 (1.0)	3 (3.1)	0	2 (2.2)
Diarrhoea	1 (1.1)	2 (2.1)	3 (3.1)	0	1 (1.0)	0	2 (2.3)	0
Dry mouth	0	0	0	1 (1.1)	2 (2.1)	2 (2.1)	2 (2.3)	1 (1.1)
Lower RTI	0	0	1 (1.0)	0	3 (3.1)	1 (1.0)	0	0

*Occurring in ≥3 patients in any group.

*OD* once daily; *BID* twice daily; *AE* adverse event; *COPD* chronic obstructive pulmonary disease; *RTI* respiratory tract infection.

The proportion of patients with serious AEs was low with all treatments and generally occurred during Period 1. Overall, a total of 21 serious AEs were reported by 19 patients, with COPD worsening being most common (reported by 5 patients). Cardiac disorder was reported in two patients (1 patient in the glycopyrronium bromide 50 μg BID group had ventricular extrasystoles, 1 patient receiving placebo had angina pectoris). One death was reported during treatment with glycopyrronium bromide 50 μg OD during period 2 but was not suspected to be related to study drug.

AEs leading to discontinuation were reported for a small proportion of patients receiving each treatment; 6 patients (6.7%), 3 patients (3.1%), 1 patient (1.1%), and 3 patients (3.1%) in the glycopyrronium bromide 12.5 μg OD, 25 μg OD, 50 μg OD, and 100 μg OD treatments, and for 1 patient (1.0%), 5 patients (5.2%) and 2 patients (2.3%) receiving glycopyrronium bromide 12.5 μg BID, 25 μg BID, and 50 μg BID, respectively, and 4 patients (4.4%) receiving placebo. The most common AE leading to discontinuation was COPD worsening. One patient receiving glycopyrronium bromide 50 μg BID had a mild headache (on Day 2 of Period 1) that lead to discontinuation and was suspected to be related to study drug.

There were no clinically meaningful changes in haematology or clinical chemistry parameter values during the study, and vital signs showed little effect of glycopyrronium bromide; in all treatment groups, mean minimum and maximum values were in the normal rage for pulse rate (40−90 bpm), diastolic blood pressure (50−90 mmHg), and systolic pressure (90−140 mmHg). Mean changes from baseline at Day 28 for ECG parameters were small in all treatment groups and not clinically meaningful overall.

## Discussion

Results from this study for trough FEV_1_ indicate that BID dosing regimens for glycopyrronium bromide produce slightly more bronchodilation compared with OD regimens for an equivalent total daily dose. However, over the course of the 24-hour dosing period, differences between OD and BID regimens, for an equivalent daily dose, were negligible, and the dose–response relationship for AUC_0-24h_ FEV_1_ was driven primarily by total daily dosage.

Dose–response results for FEV_1_ at 12 hours, FEV_1_ AUC_0-12h_ and FEV_1_ AUC_0-4h_ at steady-state showed that OD regimens provided greater improvement over placebo than BID regimens for total daily doses of 25 μg, 50 μg and 100 μg, while the reverse was true for OD versus BID regimens from 12–24 hours (see Table 2). However, over the whole day these differences tend to cancel out, thereby accounting for the lack of meaningful differences between OD and BID dosing for AUC_0-24h_. In theory, BID dosing may offer greater improvement in night-time symptoms while OD regimens provide greater improvements in day-time symptoms; the magnitude of the differences in spirometry data between the OD and BID regimens suggests that any symptomatic differences would be also be small.

The 12.5 μg BID dose produced a marginally higher improvement in trough FEV_1_ over placebo than the 50 μg OD dose at Day 28, however, the response it produced in FEV_1_ at 12 hours after 28 days of treatment over placebo (74 mL) was below the MCID of 100 mL [13]. These results therefore show that overall 50 μg daily is the lowest daily dose of glycopyrronium bromide to provide effective 24-hour bronchodilation. Of interest, similar spirometric profiles were found to those shown in the current study when aclidinium 400 μg BID was compared with tiotropium 18 μg OD; improvements from baseline in normalized FEV_1_ were significantly greater for aclidinium vs tiotropium over the last 12 hours on days 1 and 15 [14].

It has been hypothesised that OD dosing may not offer additional benefits over BID dosing in all patients; some symptomatic patients may prefer more frequent relief with a BID regimen [15]. However, simplification of dosing regimens by reducing dosing frequency has been shown to improve adherence with medication [16,17] and has been linked with higher levels of persistence [4,18,19]; increased adherence is strongly associated with reduced hospitalization and healthcare utilization costs [20,21]. It has also been suggested that bronchodilators with long durations of action provide a relatively consistent improvement in airway calibre over time, in contrast to the peaks and troughs that can arise with bronchodilators that are dosed twice-daily [22]. Assessment of the dosing regimen(s) likely to provide optimum efficacy should take into account whether adherence is likely to be compromised by increased dosing frequency, and the impact on potential non-adherence on the actual daily dose received. It is also important to remember that adherence tends to be lower in clinical practice than in a clinical trial setting [23-28].

A 24-hour duration of action, combined with a rapid onset of action, are desirable features in a novel LAMA. Phase II and III studies have consistently shown that glycopyrronium bromide 50 μg OD is a suitable dosing regimen providing significant, rapid bronchodilation that is sustained for 24-hours, and clinically meaningful efficacy [5,6,8,9,29-31]. Recently published data show that the pharmacokinetics of glycopyrronium bromide 50 μg are consistent between doses with limited systemic accumulation at steady-state after repeated once-daily inhalation [32], indicating that glycopyrronium bromide 50 μg OD is also a suitable dosing regimen from a pharmacokinetic perspective.

Spirometric measures are important endpoints and the primary means of assessing bronchodilator efficacy in clinical trials; however, symptomatic outcomes may be more meaningful to patients and physicians. Such outcomes should therefore be considered when assessing the relative efficacy of different treatment regimens. It cannot necessarily be assumed that a low-dose regimen that provides equivalent efficacy in increasing FEV_1_ will also provide equivalent efficacy on symptomatic endpoints. Indeed, a recent systematic review investigating the relationship between FEV_1_ and other outcomes in COPD found that improvements on the St. George’s Respiratory Questionnaire (SGRQ) and Transition Dyspnoea Index correlated with FEV_1_ in part, but were partly independent of changes in FEV_1_[33]. Glycopyrronium bromide 50 μg OD has been shown to effectively improve COPD symptoms and reduce exacerbations: in the Phase III GLOW1 study this treatment regimen produced a 108 mL difference in trough FEV_1_ (at 12 weeks) compared with placebo (p < 0.001), which is comparable to the difference reported in the present study (109 mL versus placebo). In addition, it provided significant improvements in dyspnoea, SGRQ and rescue medication use versus placebo, accompanied by a 31% decreased risk in moderate/severe exacerbations compared to placebo (p = 0.023) [29].

Glycopyrronium bromide was well tolerated at all doses investigated in this study. The incidence of AEs was similar in glycopyrronium bromide - and placebo-treated patients. The results support those from three recently completed Phase III studies (GLOW1, GLOW2 and GLOW3) that show glycopyrronium bromide 50 μg OD is well tolerated with a good overall safety profile [6,29,30].

Overall, this study confirmed the efficacy of glycopyrronium bromide 50 μg OD, as observed in other studies [5,8], which identified this as an appropriate dosage for further investigation in Phase III trials [6,29,30].

## Conclusions

Differences in terms of FEV_1_ AUC_0-24h_ between OD and the same total daily dose administered BID were small and not clinically relevant. Although both OD and BID dosing appear to be viable, OD dosing is associated with improved patient adherence; an important consideration when selecting the optimum dosing regimen for a novel bronchodilator. Overall, the results of this study support the efficacy and safety data from previous studies and recently completed Phase III trials (GLOW1, GLOW2 and GLOW3), which support glycopyrronium bromide 50 μg OD as a safe, effective and convenient regimen that produces consistent, 24-hour bronchodilation.

## Abbreviations

AE: Adverse event; AUC: Area under the curve; BID: Twice daily; CI: Confidence interval; COPD: Chronic obstructive pulmonary disease; ECG: Electrocardiogram; FEV_1_: Forced expiratory volume in one second; FVC: Forced vital capacity; ICS: Inhaled corticosteroids; LAMA: Long-acting muscarinic antagonist; LABA: Long-acting β_2_-agonist; MCID: Minimum clinically important difference; OD: Once daily; SGRQ: St George’s Respiratory Questionnaire.

## Competing interests

HA declares no conflict of interest. TO, DR, MG and DB are employees of Novartis Pharma AG.

## Authors’ contributions

All authors read and approved the final manuscript.

## Pre-publication history

The pre-publication history for this paper can be accessed here:

http://www.biomedcentral.com/1471-2466/12/74/prepub

## Supplementary Material

Additional file 1 **Appendix 1.** Study centers and ethics committee. Click here for file

Additional file 2 **Appendix 2.** Analysis methodology. Click here for file
