# Supplementary material for: A novel model-based approach for dose determination of glycopyrronium bromide in COPD
Source: BMC Pulm Med. 2012 Dec 8;12:74. doi: 10.1186/1471-2466-12-74 (PMC3528484; doi:10.1186/1471-2466-12-74)
Supplement: Additional file 1 — Appendix 1. Study centers and ethics committee. [file 1471-2466-12-74-S1.doc]

# Appendix 1

**List of participating centres**

**Belgium:** SGS, Life Sciences Services Clinical Research, Antwerp 2060; Longartsenpraktijk Genk, Genk 3600; Private Practice of Dr. J-B Martinot, Jambes 5100; Associatie Longziekten Hasselt, Hasselt 3500.

**Germany:** Medars GmbH, Berlin 14057; Medizinische Versorgungszentren Dr. Deckelmann, Leipzig 04207; Praxis Dr. Kampschulte, Berlin D-12165; Johannes Gutenberg-Universität, Mainz 55131; Institut für Klinische Forschung Pneumologie GmbH and Co KG, Frankfurt 60596; Medaimun GmbH, Frankfurt 60596; PAREXEL International GmbH, Berlin 14050; Momentum Pharma Services, Hamburg 22769; Harrison Clinical Research GmbH, München 80636.

**Hungary:** Dr Kenessey Albert Hospital, Balassagyarmat 2660; Drug Research Center Kft. Gyogyszervizsgalo Kozpont, Balatonfüred 8230; Pest Megyei Tudogyogyintezet, Torokbalint 2045; Kenezy Gyula Korhaz, Debrecen 4032; Jász-Nagykun-Szolnok Megyei Hetenyi Geza Korhaz-Rendelointezet, Szolnok H-5000; Orszagos Koranyi TBC es Pulmonologiai Intezet, Budapest 1529.

**India:** Metro Multispeciality Hospital, Noida Uttar Pradesh 201301; Leela More's Chest Clinic and Critical Care, Nagpur Maharashtra 440012; Indore Chest Center, Indore Madhya Pradesh 452018; PSG Hospital, Coimbatore Tamil Nadu 641004; Getwell Hospital and Research Institute, Nagpur Maharastra 400 012.

**Netherlands:** Catharina Ziekenhuis, Eindhoven 5623 EJ; Elkerliek Hospital, Helmond 5707 HA; Twenteborg Ziekenhuis, Almelo 7609 PP; Gelre Ziekenhuizen, Locatie Het Spittaal, Zutphen 7207 BA; Atrium Medisch Centrum Heerlen, Heerlen 6419 PC; Maxima Medisch Centrum, Veldhoven 5504 DB; Ziekenhuis St. Jansdal, Harderwijk 3844 DG.

**Poland:** Uniwersytecki Szpital Kliniczny nr 1 im. N. Barlickiego, Lodz 90-153; Specjalistyczna Przychodnia Lekarska "Juniperus", Izabelin 05-080; Nzoz Centrum Medyczne Szpital, Swietej Rodziny, Lodz 90-302; Specjalistyczna Praktyka Lekarska Altamed P Sliwinski i Part, Warszawa 01-456.

**Romania:** Institutul Pneumoftiziologie Marius Nasta, Bucharest 050159.

**Spain:** Clinica Mediterranea de Neurociencias, Alicante 03114; Complejo University Hospital A Coruña (antes Hospital Juan Canalejo), La Coruña 15006; Hospital del Bierzo, Ponferrada Leon 24400.

**USA:** University of California San Diego Airway Research and Clinical Trials Center, San Diego CA 92103-8415; Harbor UCLA Medical Center, Torrance CA 90502; Pinnacle Research Group, LLC, Anniston AL 36207; American Health Research, Charlotte NC 28207; California Research Medical Group, Inc., Fullerton CA 92835; Clinical Research Institute of Southern Oregon, PC, Medford OR 97504; The Clinical Research Center, St. Louis MO 63141; Palmetto Medical Research Associates, Easley SC 29640; Spartanburg Medical Research, Spartanburg SC 29303; Jasper Summit Research, Jasper AL 35501; Clinical Research Consortium, Las Vegas NV 89119.

**List of Independent Ethics Committees and Institutional Review Boards**

**Belgium:** Commisie voor Medische Ethiek, Ziekenhuisnetwerk Antwerpen 2020.

**Germany:** Llandesarztekammer Hessen, Ethik-Kommission, Im Vogelsgesang 3 60488, Frankfurt am Main.

**Hungary:** Medical Research Council Ethics Committee for Clinical Pharmacology, Budapest 1051.

**India:** Metro Ethics Review Board, Noida Uttar Pradesh 201301; BREATHE (Better Respiratory Environment And Technical Health Education Trust), Nagpur Maharashtra 440012; Apex Independent Ethics Committee, Indore Madhya Pradesh 452001; Institutional Human Ethics Committee, Coimbatore Tamil Nadu 641004; Independent Ethics Committee, Nagpur Maharashtra 440012.

**Netherlands:** METC Catharina Ziekenhuis, Eindhoven 5623 EJ; MEC Elkerliek Hospital, Helmond 5700 AB; METC- ZGT, Hengelo 7550 AM; METC Gelre Ziekenhuizen, locatie Het Spittaal, Zutphen 7207 BA; METC Atrium MC Maaslandziekenhuis, Atrium Medisch Centrum Brunssum-Heerlen, Heerlen 6401 CX; Maxima Medisch Centrum, locatie Eindhoven, Veldhoven 5504 DB; Medisch-ethische Commissie Ziekenhuis St. Jansdal, Harderwijk 3840 AC.

**Poland:** Komisja Bioetyki ds. Badan na ludziach, Uniwersytetu Medycznego w Lodzi, Al. Kosciuszki 4, Lodz NA 90-419; Komisja Bioetyczna przy Okregowej Izbie Lekarskiej w Warszawie, Ul. Pulawska 18, Warszawa NA 02-512; Komisja Bioetyczna przy Okregowej Izbie Lekarskiej w Lodzi, Ul. Czerwona 3, Lodz NA 93-005.

**Romania:** National Ethics Committee, 48 Av, Sanatescu street, Bucharest 011478.

**Spain:** CEIC CMN, Camino Viejo de Alicante-Elche s/n, Partida de Bacarot, Alicante 03114; CEIC de Galicia, Edificio Administrativo de San Lázaro, Santiago de Compostela 15073; CEIC Area de Salud de León, Hospital de Leon, Leon 24071.

**USA:** Human Research Protections Program, University of California, San Diego, La Jolla CA 92037; John F. Wolf, MD, Human Subjects Committee, Los Angeles Biomedical Research Institute, Torrance CA 90502; Quorum Review, Inc., Seattle WA 98101.
