# Supplementary material for: A novel model-based approach for dose determination of glycopyrronium bromide in COPD
Source: BMC Pulm Med. 2012 Dec 8;12:74. doi: 10.1186/1471-2466-12-74 (PMC3528484; doi:10.1186/1471-2466-12-74)
Supplement: Additional file 2 — Appendix 2. Analysis methodology. [file 1471-2466-12-74-S2.doc]

Appendix 2: Analysis methodology

The main objective of this study was to characterize the glycopyrronium bromide dose versus trough FEV_1_ relationship for OD and BID regimens. Non-linear mixed effects modeling was used for this purpose.

Based on previous glycopyrronium bromide spirometric results, the shape of the dose-response curve was assumed to be of E_max_-type:

$$E\{trough\text{ }FEV_{1}\}=E_{0}+\frac{E_{\max}\times\text{dose}}{ED_{50}+\text{dose}}$$

where the key parameters are E_max_, the maximum possible drug effect, and ED_50_, a measure of drug potency interpreted as the dose providing 50% of the maximum effect.

Within this framework key model assumptions for the present study were that regimen acts as a potency (ED_50_) modifier and that a common maximal effect (E_max_) is reached
asymptotically for both regimens.

The analysis of primary and secondary endpoints was performed using the full analysis set (FAS). The per-protocol (PP) set was also used for supportive analysis of the primary endpoint only.

# Primary Efficacy Evaluation

The key objective of the study was to characterize the glycopyrronium bromide dose versus trough FEV_1_ relationship for OD and BID regimens; as such, no formal hypothesis testing was undertaken. A statistical modelling process was used to achieve the key objective. A set of 8 candidate models was derived that describe the evolution of dose response over time. A model-averaging process was then employed to obtain response predictions as the weighted
average of individual model predictions and confidence limits derived through a simulation-based procedure. The following sections describe the modelling process in more detail.

# Primary Efficacy Variable

The primary analysis variable was trough FEV_1_ (calculated as the average of the 23 h 15 min and 23 h 45 min measurements). Even though modelling outcomes were evaluated after 28 days of treatment, trough FEV_1_ measurements collected on Day 2, Day 8, Day 15 and Day 29, from both treatment periods and from all dose groups (including once and twice daily regimens), contributed to the analysis.

# Candidate models

The modelling approach involved a priori specifying a set of candidate models that describe evolution of the response both over time (days of treatment) and dose domains. This set
included the following models, which are presented below using simplified equations
expressed in terms of total daily dose and where ‘Day’ refers to dosing day rather than
collection day (e.g. trough FEV_1_ obtained on Day 2 is assumed to be Day 1 in the analysis). Those are expected to provide similar dose-response characterization at pharmacodynamic steady state or, practically speaking, Day 28. The key differentiating feature is how each model describes evolution of the response over time, i.e. when steady state is reached. A total of 8 candidate models were considered.

***E_max_ model at steady state***: This model contrasts an E_max_ dose response at Day 1 and steady state which, based on previous glycopyrronium bromide trials, is believed to be achieved by Day 7.

$E\{\text{trough }FEV_{1}\}=E_{0}+\frac{E_{\max}\times\text{dose}}{ED_{50}\times\lambda^{I\text{(day}=\text{1)}}/\theta^{I\text{(BID)}}+\text{dose}}$ (1)

where *I*(.) is an indicator function taking value 1 for its argument and 0 otherwise, *θ* represents relative potency between BID and OD regimens. The model assumes different
potencies on Day 1 and subsequent days. This behaviour is captured through the parameter λ which acts as a scaling factor for ED_50_ on Day 1.

***Longitudinal E_max_ model***: This model attempts to characterize both time and dose dependencies of the outcome through E_max_-type relationships.

$E\{\text{trough }FEV_{1}\}=E_{0}+\frac{\text{day}}{ET_{50}+\text{day}}\times\frac{E_{\max}\times\text{dose}}{ED_{50}/\theta^{I\text{(BID)}}+\text{dose}}$ (2)

where ET_50_ can be interpreted as the time needed to reach half the maximal effect.

***E_max_ model with time-varying potency***: This model also attempts to capture evolution of the response over time using an E_max_-type relationship but, unlike the previous model, it acts as a potency modifier (through the parameter ETP50):

$E\{\text{trough }FEV_{1}\}=E_{0}+\frac{E_{\max}\times\text{dose}}{ED_{50}/(\frac{\text{day}}{ETP_{50}+\text{day}})/\theta^{I\text{(BID)}}+\text{dose}}$ (3)

This equation assumes that potency decreases over time to reach a plateau value at steady state. This is consistent with expected pharmacological principles (ED_50_ is greater on Day 1 than at steady state).

***KPD model***: The model equation is given by

$E\{\text{trough }FEV_{1}\}=E_{0}+\frac{E_{\max}\times\text{dose}}{ED_{50}/(\Theta^{I\text{(BID)}}\times\exp(-k/2^{I\text{(BID)}})\times(1-\exp(-k\times\text{day}))/(1-\exp(-k/2^{I\text{(BID)}})/2^{I\text{(BID)}})+\text{dose}}$ (4)

The model implicitly assumes that dose enters the “effect site” (lung) in bolus and is eliminated through a first-order process characterized by the rate constant *k*, which determines how fast steady state is attained (large values result in rapid steady state).

The models defined above are also represented with a sigmoidal E_max_ shape for the dose–response component, i.e. using an extra parameter γ is introduced in the dose–response component. This parameter adds flexibility to the shape of the dose response. However, it is also more difficult to identify precisely parameters from such a model, which makes it more prone to numerical issues. Having both versions, E_max_ and sigmoidal E_max_, of each model as part of the candidate set therefore helps balance risks and benefits associated with each of them. The sigmoidal models are presented below.

## Sigmoidal E_max_ model at steady state:

$E\{\text{trough }FEV_{1}\}=E_{0}+\frac{E_{\max}\times\text{dose}^{\gamma}}{(ED_{50}\times\lambda^{I\text{(day}=\text{1)}}/\theta^{I\text{(BID)}})^{\gamma}+\text{dose}^{\gamma}}$ (5)

## Longitudinal sigmoidal E_max_ model:

$E\{\text{trough }FEV_{1}\}=E_{0}+\frac{\text{day}}{ET_{50}+\text{day}}\times\frac{E_{\max}\times\text{dose}^{\gamma}}{(ED_{50}/\theta^{I\text{(BID)}})^{\gamma}+\text{dose}^{\gamma}}$ (6)

## Sigmoidal E_max_ model with time-varying potency:

$E\{\text{trough }FEV_{1}\}=E_{0}+\frac{E_{\max}\times\text{dose}^{\gamma}}{(ED_{50}/(\frac{\text{day}}{ETP_{50}+\text{day}})/\theta^{I\text{(BID)}})^{\gamma}+\text{dose}^{\gamma}}$ (7)

## Sigmoidal KPD model:

$E\{\text{trough }FEV_{1}\}=E_{0}+\frac{E_{\max}\times\text{dose}^{\gamma}}{(ED_{50}/(\Theta^{I\text{(BID)}}\times\exp(-k/2^{I\text{(BID)}})\times(1-\exp(-k\times\text{day}))/(1-\exp(-k/2^{I\text{(BID)}})/2^{I\text{(BID)}}))^{\gamma}+\text{dose}^{\gamma}}$ (8)

# Covariate adjustment

All the available trough FEV_1_ data between Day 1 and 28 were combined to fit the above models, which also included random terms to represent inter-individual (patient), inter-occasion (patient-period) and residual variability, all assumed to be normally distributed. An attempt at including inter-occasion variability on E_max_ was made but did not result in significant improvement and was therefore not retained.

Each model was further adjusted for period baseline FEV_1_ measurement (or period baseline FVC measurement, for the trough FVC analysis), FEV_1_ prior to inhalation and FEV_1_ 45 min post inhalation of ipratropium bromide (components of reversibility at Day -8 smoking status (current/ex-smoker), baseline ICS use (yes/no), and period (fixed effect). The covariates were added on to each of the dose response models described above.

# Method of implementation

The analysis was performed using the SAS procedure NLMIXED for each of the candidate models. These models were fitted using the first-order method of Beal and Sheiner [1] using NLMIXED (method = FIRO).

A model-averaging process was then implemented for those models that appropriately converge. Note that any model which converged but had a standard error divided by estimate for any dose response parameter that was greater than 5, or any standard error was missing, zero or greater than 10, then this model was not included in the model-averaging process. Models with an estimate for E_max_ of greater than 0.3 (or 0.5 for Trough FVC) or an estimate of ED50 of greater than 100 were also excluded.

# Model inference

The best-fitting model was identified using the Bayesian Information Criterion (BIC). Basing inference on the best-fitting model might be overly optimistic, however. Model averaging [2], a formal method of accounting for model uncertainty among a series of competing models, was employed to conduct more robust inference. For this purpose BIC weights were considered:

$$\frac{\exp(-0.5\times BIC_{m})}{\sum_{m} \exp(-0.5\times BIC_{m})}$$

where BIC_m_ denotes the BIC value from model m, and summation is over models in the
candidate set.

Response predictions were obtained as the weighted average of individual model
predictions using BIC weights defined above. According to this formula models that best
represent the data will carry a greater weight in the prediction. Confidence limits around model-averaged predictions were obtained using a simulation-based procedure repeating 25,000 times the following steps:

1. sample a model proportionally to BIC weights (multinomial distribution);
2. conditionally on the chosen model, sample a set of parameter values from the estimated distribution (i.e. using estimated parameter estimates and associated variance-covariance matrix from the selected model in first step) and derive the corresponding prediction.

Quantiles from the resulting distribution were used to form confidence intervals around the model-averaged predictions.

# References

1. Beal SL, Sheiner LB: **Estimating population-kinetics.** *Crit Rev Biomed Eng* 1982, **8**:195-222.
2. Hoeting JA, Madigan D, Raftery A, Volinsky CT: **Bayesian model averaging: a tutorial.** *Statist Science* 1999, **14**:382-417.
